# A Message to Health Care Providers: “A” Blood Group Is Associated with Higher Heart Disease Risk in Young Saudi Men

**DOI:** 10.3390/healthcare13222845

**Published:** 2025-11-09

**Authors:** Thamir Al-khlaiwi, Syed Shahid Habib, Abdul Manan Abdul Khalid, Hessah Alshammari, Huthayfah Al-khliwi, Abdulaziz Al-Manea, Abdulkareem Alotaibi, Salman Albadr, Feras Almasoud, Manan Alhakbany

**Affiliations:** 1 Department of Physiology, College of Medicine, King Saud University, Riyadh 11461, Saudi Arabia; talkhlaiwi@ksu.edu.sa (T.A.-k.); sshahid@ksu.edu.sa (S.S.H.); 442100659@student.ksu.edu.sa (H.A.-k.); 442100355@student.ksu.edu.sa (A.A.-M.); 442100594@student.ksu.edu.sa (A.A.); 442100266@student.ksu.edu.sa (S.A.); 442100621@student.ksu.edu.sa (F.A.); 2 Department of Blood Bank and Transfusion Medicine, King Saud University Medical City, Riyadh 11461, Saudi Arabia; aabdulkhalid@ksu.edu.sa; 3 Department of Cardiac sciences, College of Medicine, King Saud University, Riyadh 11461, Saudi Arabia; healshammari@ksu.edu.sa

**Keywords:** premature coronary artery disease, blood group, severity, prevalence, risk factor, association, Saudi population

## Abstract

Background and objectives: Given the limited number of studies evaluating the relationship of ABO blood groups and Premature coronary artery disease (PCAD) as well as the lack of relevant literature in Saudi Arabia, a study to assess the association of ABO blood groups and PCAD in Saudi population was crucial. Methods: This is a retrospective comparative study, where controls are healthy individuals and cases are divided into: patients younger than 51 years (PCAD) with confirmed coronary artery disease and patients ≥ 51 years (CAD) with confirmed coronary artery disease, whose data are retrieved from 2015 to 2022. Severity of the disease is assessed by vessel score and Gensini score. Results: We have collected a total of 1167 samples; 466 individuals served as controls (39.9%), 346 were PCAD cases (29.6%), and 355 were CAD patients (30.4%). No significant overall difference was found in ABO distribution among healthy, PCAD, and CAD individuals, although blood group A is more common in PCAD and CAD patients than in healthy controls. Among males, there is a statistically significant difference in ABO distribution across healthy, PCAD, and CAD groups, with a higher frequency of blood group A and a lower frequency of O in patients compared to controls (A = 19.7%, 28.1%, 28.4%, B = 17.5%, 19.0%, 18.6%, O = 60.0%, 48.3%, 50.2%, AB = 2.8%, 4.6%, 2.8%, *p* = 0.041, respectively). Additionally, the difference in ABO is not statistically significant between the healthy females, PCAD female patients, and CAD female patients (A = 25.5%, 31.3%, 25.7%, B = 20.7%, 13.3%, 20.0%, O = 47.2%, 53.0%, 51.4%, AB = 6.6%, 2.4%, 2.9%, *p* = 0.541, respectively). The result reveals the severity of coronary vessel occlusion in PCAD group by using Gensini score as follows: A: 52.81 ± 31.30, B: 66.94 ± 45.57, O: 43.06 ± 32.95, AB: 49.00 ± 49.40 with *p* value = 0.131. Conclusions: The present findings suggest that higher frequency of blood group “A” was found among male patients with PCAD and CAD compared to other blood groups. In addition, blood group “O” is less associated with male PCAD and CAD in Saudi population. Identification of ABO blood groups might assist in the genetic screening as well as guiding prophylaxis for premature CAD.

## 1. Introduction

One neglected category of coronary artery disease (CAD) is premature coronary artery disease (PCAD). It is defined as CAD occurring in younger males and females; different studies have used different cut-offs varying from 45 to 65 years of age [1,2,3]. CAD usually affects elderly people. However, it is estimated that 4–10% of CAD patients are younger than 45 years [4]. More specifically, a study conducted in 2014 revealed that PCAD prevalence was 11% in the Middle East [5] while the prevalence in Africa is 9.7%, in North America is 4%, and in Western Europe is 2.7% [6].

Astonishingly, a study conducted in Saudi Arabia showed that in 49% of the total cases of ST-elevation myocardial infarction (STEMI) patients were under 45 years of age [7]. Another study in Saudi Arabia observed that 16.7% of patients presenting with acute coronary disease were younger than 45 years [8]. Compared to a population in an adjacent geographical region (Iran), a study observed that 5.4% of 2028 patients presenting with acute STEMI were younger than 40 years [9].

Development of PCAD is governed by multiple risk factors; some are traditional risk factors, while others are nontraditional risk factors related to genetic predisposition. Smoking showed to be the most common cardiovascular risk factor (60.8%) associated with PCAD according to a study conducted in the United States in 2020 followed by hypertension (52.8%) and family history of CAD (39.8%) [10]. It is found that the distribution of risk factors differs between patients with PCAD and CAD. In a systematic review and meta-analysis performed in Australia in 2021, opium use, family history of CAD, smoking, and dyslipidemia were found to be more common in PCAD patients, whereas hypertension, obesity, diabetes mellitus, and sedentary lifestyle were shown to be more prevalent among those who have CAD [1]. Genetic susceptibility is the primary contributing factor to the pathogenesis of PCAD as suggested by a study performed in China [11]. Ethnicity may play a role in the inheritance of variants which contribute to PCAD [11]. The effects of genetic predilection are augmented by traditional cardiovascular risk factors, such as smoking, diabetes mellitus, hypertension, obesity, and hyperlipidemia. Therefore, high hypertension, blood glucose, and body mass index (BMI) are found in patients with PCAD relative to normal healthy individuals [11]. Diabetes mellitus was found to be the most common risk factor for PCAD in the Iranian population followed by dyslipidemia, history of CAD in the family, smoking and hypertension [12]. In Saudi Arabia, history of smoking and substance abuse are the risk factors that were significantly higher in PCAD compared to CAD patients with a prevalence of 52% and 3%, respectively [7].

ABO blood groups have been linked to CAD [1]. For instance, studies conducted in China and India revealed that the severity of CAD is affected by blood group A [13,14]. Also, a study found that non-O blood group patients had approximately double the risk of developing CAD as well as having more stenosis in the carotid arteries [15]. Moreover, a study investigated the relation between ABO and peripheral artery disease (PAD), and the coexistence of CAD revealed a significant association between ABO blood groups and both the severity of PAD and the association of CAD, and shows that non-O blood groups are linked to more severe forms of these conditions [16]. Venous thromboembolism had also been correlated with ABO blood groups in a study showed that the non-O blood groups are more associated with thromboembolism [17]. Recently, blood groups B and AB were found to be correlated with stent thrombosis [18].

On the other hand, the association of ABO blood groups with PCAD is still controversial [18,19,20,21].

Since there are few studies evaluating the relationship of ABO blood groups and PCAD worldwide, the frank discrepancy in the literature regarding the association, as well as the lack of relevant literature in Saudi Arabia, the aim of this study is to assess the association of ABO blood groups and PCAD as well as the severity of the disease in Saudi population.

## 2. Materials and Methods

The present study is a retrospective comparative study conducted in Riyadh, Saudi Arabia. Data of the present study were collected from patient’s electronic medical records from King Saud University Medical City (KSUMC) from the period of 2015 to 2022. Data regarding demographic characteristics (age, gender, height, weight, BMI) and patients’ diagnosis were collected from patients’ electronic medical records (eSIHI). Data regarding subject’s ABO blood groups were collected from the blood bank system (Hematose).

To assess the severity of vessel involvements, we used well known scores that are widely used in clinical practice: Vessel score and Gensini score. Vessel score measures the number of main vessels—left anterior descending artery (LAD), left circumflex artery (LCx), and right coronary artery (RCA)— with significant stenosis (>50%). According to the number of vessel involvements, patients were classified as: single; 1, double; 2, and triple; 3 vessel disease. If multiple lesions were found in the same artery, it was considered as single vessel disease. In addition, Gensini score was used to assess the severity of the occlusion. Gensini score is a well-known assessment measurement which is widely used in similar research. A zero score means no occlusion. The Gensini score is a reliable and validated estimate of the occlusion severity of coronary arteries by objectively evaluating the localization, extent, and percent of arterial occlusion. Due to the missing data, only 98 angiographic reports were found in the system for PCAD patients.

The study group was divided into: (PCAD) patients younger than 51 years of age with confirmed coronary artery disease, and (CAD) patients ≥ 51 years of age. Both groups (PCAD and CAD) underwent selective coronary angiography using the routine procedure in the hospital (right femoral artery approach). Coronary imaging was assessed using right and left oblique views with cranial and caudal positions. Involvement of at least one of the primary coronary arteries (LM, LAD, LCx, RCA) can be considered as inclusion criteria. PCAD population include individuals with acute coronary syndrome, or only individuals undergoing planned diagnostic testing for suspected chronic coronary syndrome. All the tests determined during the same hospitalization during which the coronary angiography was performed. The control group was normal healthy individuals who donated their blood in a blood bank and were examined by a primary physician to be clear from cardiovascular disease. We compared the demographics and biochemical profile in all 3 groups (control, PCAD, and CAD) based on different blood groups. The age, weight and BMI differences were non-significant among ABO groups. Control subjects were matched to the cases by age and BMI. Since there are differences in the cut-offs that are used to define PCAD varying from 45 to 65 years of age, we used 50 years of age as our cut-off between PCAD and CAD [1,2,3].

Inclusion criteria: Saudi males and females older than 17 years of age with coronary artery disease confirmed by angiography while the exclusion criteria: Non-Saudis (to reduce ethnic variation), patients with hematological disorders, patients with variant angina, and patients with chronic kidney disease.

The study was approved by the Institutional Review Board (IRB), College of Medicine, King Saud University (No. E-24-8483) in accordance with the Declaration of Helsinki. The study maintained the privacy and confidentiality of all the information of the participants. Participants’ anonymity will be assured.

### Statistical Analysis

Data were analyzed using IBM SPSS Statistical software for Windows version 26.0 (IBM Corp., Armonk, NY, USA). Descriptive statistics (frequencies, percentages, mean and standard deviation) were used to describe the categorical and quantitative variables. For more than two groups, ANOVA was used for income variables (age, BMI, gender, lipid profiles, HbA1c) to compare the mean values in relation to the outcome study variables (ABO distribution) in a three-group comparison; Chi-square test was used to compare categorical data. Post hoc Bonferroni test was used to compare between the subgroups. All continuous variables were checked for normality using the Kolmogorov–Smirnov test. A *p*-value of ≤0.05 and 95% confidence intervals were used to report the statistical significance and precision of the results. Multivariate binary logistic regression was performed for predictive value of ABO blood groups considering O as reference category and HbA1c BMI and dyslipidemia as covariates.

## 3. Results

In this study, we collected a total of 1167 samples—466 individuals served as controls (39.9%), 346 were PCAD cases (29.6%), and 355 were CAD patients (30.4%)—to compare ABO bloods group. Table 1 represents baseline characteristics according to blood groups in PCAD patients. It shows the mean age of PCAD patients with various blood groups as follows: A: 42.95 ± 6.30, B: 43.77 ± 5.38, O: 44.16 ± 5.37, AB: 41.29 ± 9.20 with *p* value = 0.169, while the mean BMI for the PCAD patients according to blood groups: A: 31.34 ± 12.82, B: 31.21 ± 6.56, O: 29.91 ± 6.91, AB: 30.86 ± 7.13 with *p* value = 0.575. In addition, Table 1 shows the severity of vessel occlusion by using Gensini score in different blood groups as follows: A: 52.81 ± 31.30, B: 66.94 ± 45.57, O: 43.06 ± 32.95, AB: 49.00 ± 49.40 with *p* value = 0.131. Although not statistically significant, O blood group shows low disease severity score among PCAD patients. It also shows that the distribution of sex, height, weight, LDL, HDL, and TG do not differ significantly between the different ABO blood groups in PCAD patients. However, HbA1c shows statistically significant difference among the blood groups, which is higher in PCAD patients with AB blood group (A = 7.64 ± 2.19 vs. B = 7.46 ± 1.95 vs. O = 7.74 ± 2.46 vs. AB = 10.70 ± 2.28, *p* = 0.008). Table 2 and Table 3 describe the baseline characteristics of the CAD and healthy groups, respectively.

**Figure 1:**
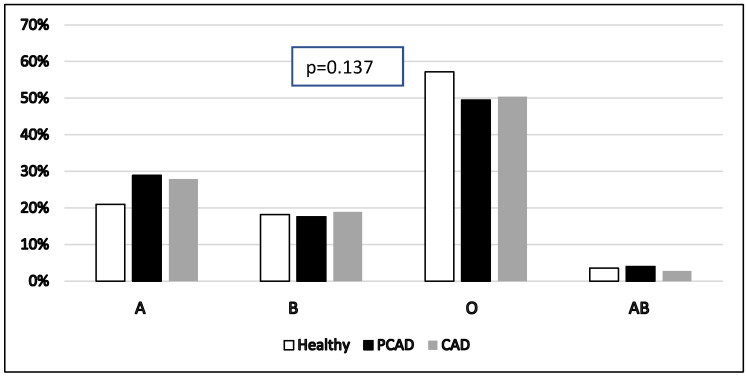
Comparison of different ABO blood groups in total sample of healthy, PCAD, and CAD patients (N = 1167). *X*-axis represents various ABO blood groups while *Y*-axis represents its percentage. Pearson’s chi-square tests were used. PCAD: premature coronary artery disease. CAD: coronary artery disease.

Figure 1 shows a comparison of percentages of different ABO blood groups among healthy, PCAD, and CAD groups (A = 21.0%, 28.9%, 27.9%, B = 18.2%, 17.6%, 18.9%, O = 57.2% vs. 49.5%, 50.4%, AB = 3.6%, 4.0%, 2.8%, respectively). There is no statistically significant difference among healthy individuals, PCAD patients, and CAD patients regarding ABO blood group distribution with a *p* = 0.137. However, one can notice that the relative percentage of “A” blood group is higher among PCAD patients and CAD patients as opposed to other blood groups which are relatively lower among cases.

**Figure 2:**
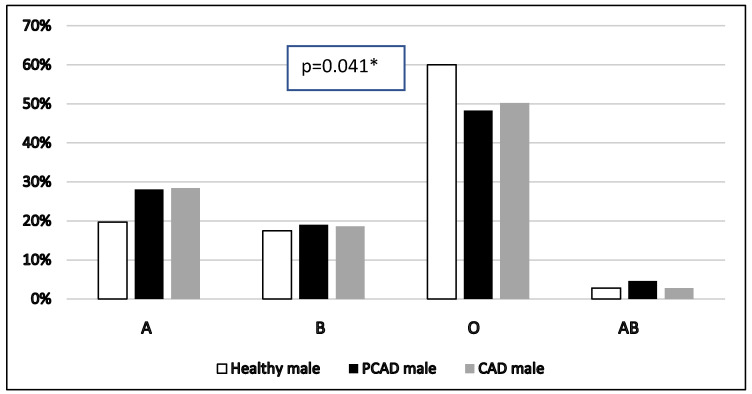
Comparison of ABO blood groups among healthy male, PCAD male, and CAD male, (n = 908). *X*-axis represents various ABO blood groups while *Y*-axis represents its percentage. Pearson’s chi-square tests were used. * significant. PCAD: premature coronary artery disease. CAD: coronary artery disease.

**Figure 3:**
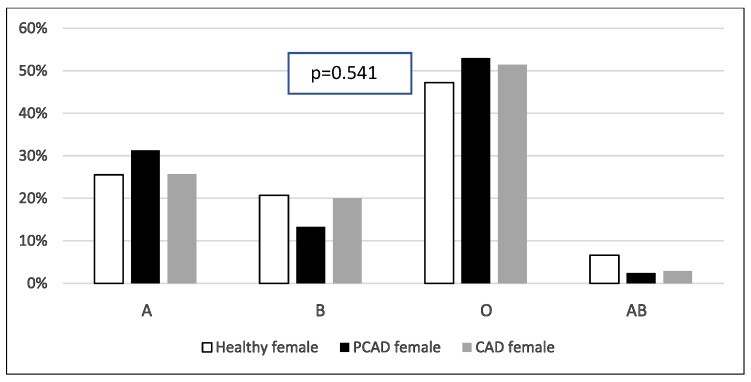
Comparison of ABO blood groups among healthy female, PCAD female, and CAD female subjects (n = 259). *X*-axis represents various ABO blood groups while *Y*-axis represents its percentage. Pearson’s chi-square tests were used. PCAD: premature coronary artery disease. CAD: coronary artery disease.

As shown in Figure 2 and Figure 3, there is a statistically significant difference in blood group distribution among healthy males, PCAD male patients, and CAD male patients (A = 19.7%, 28.1%, 28.4%, B = 17.5%, 19.0%, 18.6%, O = 60.0%, 48.3%, 50.2%, AB = 2.8%, 4.6%, 2.8%, *p* = 0.041, respectively). However, the difference is not statistically significant between healthy females, PCAD female patients, and CAD female patients (A = 25.5%, 31.3%, 25.7%, B = 20.7%, 13.3%, 20.0%, O = 47.2%, 53.0%, 51.4%, AB = 6.6%, 2.4%, 2.9%, *p* = 0.541, respectively). Furthermore, the frequency of “A” blood group is higher among males with PCAD and CAD compared to healthy males while “O” blood group is higher in healthy subjects when compared to cases.

Table 4 shows the Association of risk factors with different ABO blood groups in PCAD patients, a statistically significant difference was found among ABO blood groups regarding smoking (A = 30.8% vs. B = 12.8% vs. O = 49.4% vs. AB = 7.1%, *p* = 0.047). However, other risk factors do not differ significantly among different ABO blood groups in PCAD patients.

Multivariate binary logistic regression was performed for predictive values of ABO blood groups considering O as a reference category and HbA1c BMI and dyslipidemia as covariates. It shows that in male patients A blood group has OR of 1.9 times in relation to coronary artery diseases with a *p* value = 0.029 (Table 5).

Table 6 shows that there is no consistent relationship between ABO blood groups and the severity of the disease using vessel score (*p* = 0.341). Although not statistically significant, there is a consistent decrease in the frequency of subjects with the “O” blood group with a higher number of significantly stenosed vessels (One vessel occlusion: 20 (39.2%), two vessels occlusion: 18 (35.3%), and three vessels occlusion: 13 (25.5%)).

Table 7 shows a comparison between blood group and low and high Gensini score in PCAD patients. Although not statistically significant, there is a decrease in the frequency with the O blood group with high Gensini score (low: 56.9% vs. high: 43.1%). At the same time, “A” and “B” blood groups show increased frequency in high Gensini score in PCAD patients (low: 40.7% vs. high: 59.3%, low: 37.5% vs. high: 62.5%, *p* = 0.274, respectively).

**Figure 4:**
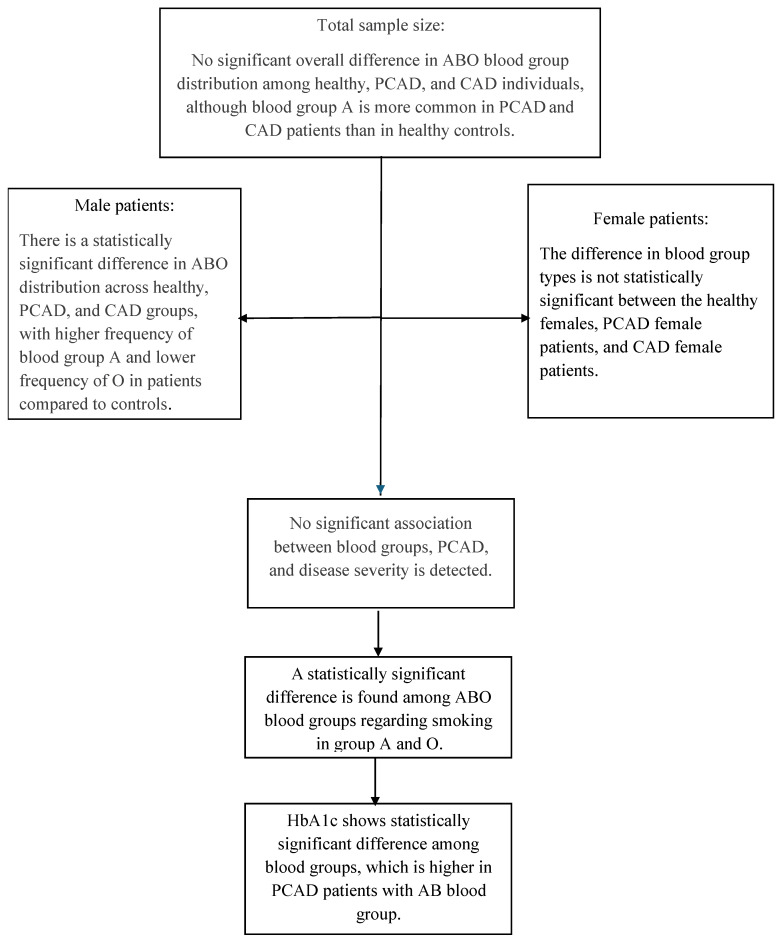
Description summary of the association of ABO blood groups and PCAD.

Figure 4 summarizes the correlation between ABO blood groups, PCAD, and gender differences.

## 4. Discussion

The present study reveals a higher frequency of blood group “A” among male patients with PCAD and CAD compared to other blood groups. In addition, blood group O is associated with less prevalence of PCAD and CAD in the Saudi male population. Our results are in concomitance with a study conducted in Taiwan in 2012 that revealed that ABO blood groups are associated with PCAD, with blood group “A” being overrepresented in the group sample constituting 39.7% of the total study sample size, followed by blood group O (30.1%), blood group B (26.5%), and blood group AB (3.7%) which appeared to be different from the distribution of the Taiwanese normal population (O, 42.6%; A, 24.0%; B, 27.1%; AB, 6.2%). However, the study was conducted with both genders [19]. On the other hand, a study investigated the relationship between ABO blood groups and PCAD in the Iranian population found no association between ABO and PCAD with distribution of ABO blood groups similar to that of a normal Iranian population (O, 35.5%; A, 32.1%; B, 24.8%; AB, 7.6%) which is not in agreement with our study, especially in male groups [20].

Regarding female patients, a study that evaluated risk factors associated with myocardial infarction (MI) in females younger than 50 years of age found that blood group “A” was significantly associated with MI, which we did not find in our results. This study however only included women, and is not a recent study [21]. Another study conducted in Turkey in 2008, observed that patients with blood group “A” were significantly younger than patients with non-A blood groups [22]. Furthermore, an observational study performed in Italy showed that ABO blood groups have a prognostic value with non-O blood groups being correlated to the death of patients less than 65 years of age due to cardiovascular disease specifically in female patients. This study comes to agreement with our findings that blood group “A” is more associated with PCAD and CAD while blood group “O” is less associated. ABO blood groups were even more superior to diabetes mellitus linked to cardiac mortality [23]. This study was performed on an exclusively Italian Caucasian population which limits the use of the study’s results on other populations. A study conducted in Croatia involving 646 individuals with CAD found that patients with AB blood group presented with CAD significantly younger than those with non-AB blood groups. In addition, males younger than 50 years were over-represented among CAD patients with AB blood groups [24]. Even though we did not find a correlation between ABO blood groups and female PCAD patients (this could be due to the small size of female PCAD patients which was expected), we observed in previous study that female patients have a higher risk of cardiovascular diseases due to a higher systolic blood pressure and LDL [25]. In addition, we recently observed that the prevalence of anterior ST elevation is higher in female compared to male PCAD patients [26]. All these findings suggest a more complicated mechanism of injury for ABO blood groups, taking into consideration the impact of hormonal differences between gender in younger populations. However, A larger scale study is of great value in this regard to confirm the findings.

Our results did not reveal a correlation between ABO blood groups and severity of PCAD. However, there is a clear pattern that 3 vessel disease frequency is lower among patients with blood group O, suggesting a negative correlation. The fact that there was no statistically significant association between ABO blood groups and the number of significantly stenosed vessels could be attributed to the low sample size of patients with calculated vessel score. When using Gensini score, the prevalence of severe stenosed vessels was higher in “A” and “B’ blood groups even though it was not significant while the prevalence of less stenosed vessels was observed in O blood group. We have not found a satisfactory number of studies investigating the nexus between the aforementioned variables. However, a study conducted in Taiwan found no association between ABO blood groups and severity of PCAD using the number of markedly stenosed vessel (>50%) as a marker of severity, which is similar to our findings [19].

The pathogenic mechanism underlying the relationship between the ABO blood groups and PCAD is still poorly understood. It is worth mentioning that the ATP-binding cassette 2 (ABCA2) gene, which has a role in cholesterol metabolism, and the ABO gene, are both located on chromosome 9q34 [22]. Blood group antigens A and B are expressed on the red blood cells as well as on other cells such as platelets and vascular endothelial cells. The higher levels of the von Willebrand factor were mentioned in the literature as the cause of increased risk of thrombosis in non-O blood groups [23]. On the other hand, it has been observed that O blood group has a 30–40% lower von Willebrand factor concentration [27]. Several mechanisms have been proposed including von Willebrand factor in O blood group enhances the function ADAMTS13 proteolysis, reduction in von Willebrand factor activity on platelets, and the ABO(H) carbohydrate of von Willebrand factor [28]. Also, a genome-wide association study showed that an ABO genetic variant linked to blood group O decreases the risk of myocardial infarction in patients with angiographically confirmed CAD [29,30]. Hence, blood group antigens may alter the risk of PCAD by affecting the levels of hemostasis and inflammatory proteins in circulation by increasing the levels of several inflammatory cytokines such as tumor necrosis, factor-alpha, and interleukin-6 [29,30].

Even though there were no differences in LDL-C and HDL-C among different ABO blood groups, the results of our suggest that an increased risk of thrombosis is more likely than atheroma formation in explaining the association between the ABO blood groups and PCAD. Our findings reveal that blood group O remained less associated with PCAD despite the high association with smoking. Moreover, we found a statistically significant association between HbA1c and blood group AB. Experimental studies are needed to elucidate the association between HbA1c and blood group AB.

Recently, we observed in a meta-analysis study conducted in Saudi Arabia (2024) that a mortality prevalence of PCAD patients ranges from 2 to 8% which is similar to the prevalence in older patients which was 2–10% [31]. In addition, severity among various age PCAD groups was found to be not similar [32]. At the same time, high prevalence of lack of knowledge was also observed in the public regarding PCAD and its risk factors [33]. This astonishing finding needs more attention and careful prophylactic plans for the young generation. As a prophylactic approach, physicians should monitor the development of calcification processes within coronary arteries in at risk patients and provide preventive measures as found recently [34]. Therefore, a larger, prospective, multicenter, and nationwide study, in which patients are stratified according to the ABO blood group genotypes, plasma levels of lipids, inflammatory markers, and hemostasis markers, would be essential to clarify the underlying mechanisms by which the ABO blood groups influence the risk of PCAD in the Saudi population.

### Strengths and Limitations

In addition to the limitations associated with the retrospective study design, the other limitations of the study were the single-center design of the study, lack of complete angiographic findings, incomplete lipid profile data, and a lack of detailed genotype information of the ABO alleles. In addition, due to the retrospective nature of the study much of the baseline characteristic information of our control subjects was lacking. Also, some ABO subgroups were small in size which might affect the comparison analysis between groups but as mentioned before, we collected all the patients affected with PCAD during 2015–2022 and this is the sample size that we found. To our knowledge our study is the first to assess the relationship between PCAD and ABO blood groups in Saudi Arabia and its correlation to the severity of the disease.

## 5. Conclusions

The present findings suggest that a higher frequency of blood group “A” was found among male patients with PCAD and CAD compared to other blood groups. In addition, blood group “O” is associated with less prevalence of male PCAD and CAD in a Saudi population. Identification of ABO blood groups might assist in genetic screening as well as guiding prophylaxis for premature CAD.

## Figures and Tables

**Table 1:** Baseline characteristics according to blood groups in PCAD patients (n = 346).

Parameter	Total Size	A	B	O	AB	*p*-Value
N (%)	346 (100)	100 (28.9)	61 (17.6)	171 (49.4)	14 (4.0)	0.491
Male, n (%)	263 (76.1)	74 (28.1)	50 (19.0)	127 (48.3)	12 (4.6)	
Female, n (%)	83 (23.9)	26 (31.3)	11 (13.3)	44 (53.0)	2 (2.4)	
Age (years)	43.04 ± 6.56	42.95 ± 6.30	43.77 ± 5.38	44.16 ± 5.37	41.29 ± 9.20	0.169
Height (cm)	168.65 ± 8.50	167.01 ± 10.90	168.98 ± 8.41	167.48 ± 8.25	171.14 ± 6.47	0.274
Weight (kg)	87.14 ± 19.82	85.36 ± 17.24	88.84 ± 19.36	84.01 ± 20.38	90.36 ± 22.33	0.299
(BMI) kg/m^2^	80.83 ± 8.35	31.34 ± 12.82	31.21 ± 6.56	29.91 ± 6.91	30.86 ± 7.13	0.575
LDL (mmol/L)	2.65 ± 1.20	2.21 ± 1.38	2.66 ± 1.13	2.82 ± 1.17	2.93 ± 1.14	0.632
HDL (mmol/L)	1.06 ± 0.28	1.05 ± 0.21	1.05 ± 0.28	1.08 ± 0.40	1.07 ± 0.26	0.915
TG (mmol/L)	1.66 ± 1.03	1.66 ± 1.06	1.62 ± 1.05	1.60 ± 1.05	1.79 ± 0.97	0.906
HbA1c	8.38 ± 2.22	7.64 ± 2.19	7.46 ± 1.95	7.74 ± 2.46	10.70 ± 2.28 **	0.008 *

Data are represented as mean and standard deviation. * significance by using ANOVA and Pearson’s chi-square test. ** is significant by using post hoc Bonferroni test. LDL: low-density lipoprotein; HDL: high-density lipoprotein; BMI: body mass index; HbA1c: glycosylated hemoglobin; TG: Triglyceride.

**Table 2:** Baseline characteristics according to blood groups in CAD patients (n = 355).

Parameter	Total Size	A	B	O	AB	*p*-Value
N (%)	355 (100)	99 (27.9)	67 (18.9)	179 (50.4)	10 (2.8)	0.275
Male, n (%)	285 (80.2)	81 (28.4)	53 (18.6)	143 (50.2)	8 (2.8)	
Female, n (%)	70 (19.8)	18 (25.7)	14 (20.0)	36 (51.4)	2 (2.9)	
Age (years)	57.22 ± 10.49	54.95 ± 10.30	57.98 ± 11.94	59.08 ± 9.54	56.89 ± 10.19	0.798
Height (cm)	159.41 ± 6.65	160.43 ± 7.55	159.87 ± 9.88	162.32 ± 6.65	155.04 ± 2.54	0.090
Weight (kg)	74.99 ± 15.27	76.54 ± 13.23	74.55 ± 15.32	71.81 ± 17.65	77.09 ± 14.87	0.299
(BMI) kg/m^2^	29.79 ± 6.70	28.54 ± 9.76	29.65 ± 7.09	29.91 ± 6.91	31.09 ± 3.06	0.212
LDL (mmol/L)	2.62 ± 1.42	2.77 ± 1.43	2.68 ± 2.09	2.02 ± 1.11	3.01 ± 1.06	0.656
HDL (mmol/L)	0.82 ± 0.57	0.98 ± 0.51	0.78 ± 0.01	0.96 ± 0.87	0.56 ± 0.89	0.713
TG (mmol/L)	1.74 ± 1.35	1.89 ± 1.23	1.54 ± 1.22	1.65 ± 1.98	1.89 ± 0.99	0.093
HbA1c	7.07 ± 3.18	6.88 ± 3.14	7.09 ± 2.55	6.87 ± 3.19	7.45 ± 3.87	0.843

Data are represented as mean and standard deviation. LDL: low-density lipoprotein; HDL: high-density lipoprotein; BMI: body mass index; HbA1c: glycosylated hemoglobin; TG: Triglyceride.

**Table 3:** Baseline characteristics according to blood groups in healthy subjects (n = 466).

Parameter	Total Size	A	B	O	AB	*p*-Value
N (%)	466 (100)	98 (21.0)	85 (18.2)	266 (57.2)	17 (3.6)	0.154
Male, n (%)	360 (77.2)	71 (19.7)	63 (17.5)	216 (60.0)	10 (2.8)	
Female, n (%)	106 (22.8)	27 (25.5)	22 (20.7)	50 (47.2)	7 (6.6)	
Age (years)	42.77 ± 5.78	41.93 ± 5.34	42.72 ± 6.23	41.22 ± 3.44	45.21 ± 8.11	0.219
Height (cm)	168.83 ± 8.68	169.23 ± 11.32	166.56 ± 7.54	169.12 ± 6.11	170.43 ± 9.76	0.467
Weight (kg)	87.19 ± 19.54	83.45 ± 18.12	89.32 ± 16.32	86.11 ± 22.65	89.89 ± 21.07	0.154
(BMI) kg/m^2^	31.57 ± 7.47	32.78 ± 10.34	31.45 ± 7.11	30.09 ± 5.89	31.98 ± 6.55	0.768
LDL (mmol/L)	1.51 ± 1.01	1.09 ± 1.04	1.76 ± 1.09	1.87 ± 1.34	1.32 ± 1.78	0.256
HDL (mmol/L)	0.87 ± 0.28	0.99 ± 0.54	0.72 ± 0.48	1.01 ± 0.09	0.76 ± 0.04	0.121
TG (mmol/L)	1.29 ± 0.99	1.09 ± 1.32	1.43 ± 1.12	1.12 ± 1.09	1.54 ± 0.45	0.414
HbA1c	5.37 ± 2.43	5.23 ± 2.11	5.46 ± 1.43	5.03 ± 3.11	5.78 ± 3.09	0.175

Data are represented as mean and standard deviation. LDL: low-density lipoprotein; HDL: high-density lipoprotein; BMI: body mass index; HbA1c: glycosylated hemoglobin; TG: Triglyceride.

**Table 4:** Association of risk factors with different ABO blood groups in PCAD patients (n = 346).

Parameter	A	B	O	AB	*p*-Value
Diabetes mellitus, n = 170 (%)	45 (26.5)	27 (15.9)	92 (54.1)	6 (3.5)	0.396
Hypertension, n = 163 (%)	45 (27.6)	26 (16.0)	86 (52.7)	6 (3.7)	0.690
Hyperlipidemia, n = 135 (%)	34 (25.2)	30 (22.2)	64 (47.4)	7 (5.2)	0.204
Smoking, n = 156 (%)	48 (30.8)	20 (12.8)	77 (49.3)	11 (7.1)	0.047 *
Family history of PCAD, n = 55 (%)	14 (25.5)	6 (10.9)	32 (58.1)	3 (5.5)	0.204

Data are represented as frequency and percent. Pearson’s chi-square tests were used. * significant.

**Table 5:** Multivariate binary logistic regression models for coronary artery disease vs. healthy subjects (N = 1167).

Variable	Level	OR	95% Confidence Limits	*p*-Value
ABO blood groups for males	O	Ref		0.029
	A	1.957	1.243–3.082	
	B	1.035	0.613–1.747	
	AB	1.479	0.565–3.867	
ABO blood groups for females	O	Ref		0.931
	A	1.02	0.676–1.540	
	B	0.945	0.598–1.494	
	AB	0.756	0.301–1.901	

**Table 6:** Association between ABO blood groups and the severity of PCAD, graded by the number of significantly stenosed vessels (n = 98).

ABO Blood Groups	Number of Significantly (>50%) Stenosed Vessels (1)	Number of Significantly (>50%) Stenosed Vessels (2)	Number of Significantly (>50%) Stenosed Vessels (3)	*p* Value
A, n (%)	7 (25.9)	12 (44.4)	8 (29.7)	0.341
B, n (%)	6 (37.5)	2 (12.5)	8 (50.0)	
O, n (%)	20 (39.2)	18 (35.3)	13 (25.5)	
AB, n (%)	2 (50.0)	1 (25.0)	1 (25.0)	

Data are represented as frequency and percent. Pearson’s chi-square tests were used.

**Table 7:** Comparison of blood groups and low and high Gensini score in PCAD patients (n = 98).

ABO Blood Groups	Low Gensini Score (<44) (n = 49)	High Gensisi Score (≥44) (n = 49)	*p* Value
A, n (%)	11 (40.7)	16 (59.3)	0.274
B, n (%)	6 (37.5)	10 (62.5)	
O, n (%)	29 (56.9)	22 (43.1)	
AB, n (%)	3 (75.0)	1 (25.0)	

Data are represented as frequency and percent. Pearson’s chi-square tests were used.

## Data Availability

The complete data and materials used and analyzed in the current study are available from the corresponding author upon reasonable request.
